# The Effects of Increasing Penalties in Drunk Driving Laws—Evidence from Chile

**DOI:** 10.3390/ijerph17218103

**Published:** 2020-11-03

**Authors:** Andrés García-Echalar, Tomás Rau

**Affiliations:** 1 Facultad de Ingeniería y Ciencias Aplicadas, Universidad de los Andes, Chile, Santiago 7620086, Chile; agarcia@uandes.cl; 2 Instituto de Economía, Pontificia Universidad Católica de Chile, Santiago 7820436, Chile

**Keywords:** drunk driving law, increasing penalties, car accidents, injuries and deaths

## Abstract

This paper analyzes Chile’s drunk driving laws and their effects on car crashes, injuries, and deaths. There were two policy changes. While the 2012 law increased license suspension penalties and decreased the legal blood alcohol limits for drivers, the 2014 law only increased sanctions, including at least one year of actual imprisonment for drunk driving implicated in car crashes with severe injury or death. We use a rich data set of countrywide administrative records that permit us to identify direct measures of alcohol-related accidents, including fatalities and injuries. We also have access to blood alcohol tests to assess whether the laws affected drivers’ alcohol consumption. Using count data models and a rich set of covariates, including police stops and gasoline sales, we find a short-run decrease in accidents and injuries for the 2012 law and a sustained decline in these outcomes for the 2014 law. Neither intervention has an effect on deaths. There is a marginal decline in alcohol consumption after the enactment of both legal changes. However, while the 2012 law only affects male drivers, the 2014 law affects both males and females. No reductions in alcohol intake are found for heavy drinkers.

## 1. Introduction

In 2016, car accidents were the eighth leading cause of death, causing 1.35 million fatalities worldwide, with a rate of 18.2% per 100,000 inhabitants [1]. It has been widely reported that the driver having consumed alcohol increases both the risk of car accidents and the risk that they will cause severe injury or death [2,3,4]. Globally, 21.8% of all deaths from vehicular accidents are related to alcohol, resulting in approximately 306,002 deaths [5].

Many countries have implemented drunk driving laws to reduce alcohol-related car crash deaths, and Chile is no exception. In the last decade, there have been two significant legal changes: the Zero Tolerance Law (ZTL) in 2012 and Emilia’s Law (EML) in 2014. While the ZTL decreased the grams of alcohol in the blood legally permissible for driving and increased the driver’s license suspension, EML added at least one year of actual imprisonment for drunk driving responsible for severe injury or fatal car crashes. EML did not change the limits on the amount of alcohol present in the driver [6].

While most of the empirical evidence has focused on lowering blood alcohol limits for driving on car accidents and deaths [7,8,9,10,11,12,13], less is known about the effects of increasing penalties, such as actual imprisonment for drunk driving, on the abovementioned outcomes [14,15,16,17]. As noted by Weinrath and Gartrell [18], “we actually know little about the deterrence effect of imprisonment.” More important, most of the empirical evidence on the effects of imprisonment on traffic fatalities finds nonsignificant effects.

In this paper, we assess the short- and medium-run effects of Chile’s ZTL and EML on vehicle accidents, injuries, and deaths with a special focus on the deterrence effect of including prison sentences for drunk driving. As argued by Hansen [19], increasing the marginal punishment when the driver’s blood alcohol content (BAC) level is higher may cause the driver to internalize the external costs of driving while intoxicated. This is especially true for drivers with very high alcohol levels for whom the external costs are higher, since such cases are more likely to lead to severe injuries or deaths. However, Bouffard, Niebuhr, and Exum [20] show that there is not always a deterrence effect of higher sentences for this type of crime. Other authors have expressed contrasting views about the effectiveness of prison sentences in the past. While some short-term effects were found by Nichols and Ross [15] and Kenkel [16], none were found by Ross and Klette [17] or Ross, McCleary & Lafree [14].

We have access to a rich dataset on car accidents and their causes to study the direct effects of each law on alcohol-related crashes. Our data include detailed information on injuries and deaths associated with each accident. We also have access to blood alcohol tests to assess the effects of these laws on drinking and driving.

The empirical approach follows Otero and Rau [13]. We implement negative binomial regressions to assess the effects of these laws on alcohol-related accidents, injuries, and deaths. Generalized linear models and censored quantile regressions are estimated to evaluate the BAC changes associated with the two legal changes.

We found statistically significant reductions in alcohol-related accidents and injuries associated with the ZTL, but they were short-lived. On the other hand, EML is associated with a permanent reduction in alcohol-related accidents and injuries. Neither of the laws affects traffic fatalities. For the ZTL, there are associated reductions in male drivers’ alcohol intake only; for EML, reductions are present for males and females. However, the effects do not reach higher quantiles of the BAC distribution.

## 2. Institutional Background

In Chile, car crashes are the second leading cause of death of young people between 15 and 25 years old and the first among children under 15 years old [21]. The number of road deaths was nearly 1950 in 2018, with a rate of 10.5 deaths per 100,000 inhabitants and an estimated cost of traffic crashes of approximately US $ 6 billion for the state [22]. Moreover, for the 2009–2017 period, approximately 30,000 drivers were apprehended every year for driving under the influence of alcohol (DUI) or while intoxicated, causing nearly 5140 traffic accidents, 5070 injured, and 165 deaths [23].

To decrease the social losses caused by DUI and drunk driving, Chile’s senate approved two legal changes regarding drunk driving in the last decade. On 15 March 2012, Law 20, 580 (the ZTL) was enacted, lowering the legal driving BAC level and increasing the license revocation period for DUI and drunk driving. The permitted BAC was reduced from 0.05 to 0.03 g/dL, and DUI was set between 0.03 and 0.079 BAC (instead of the former 0.05–0.099 BAC range). The starting threshold for drunk driving was reduced from 0.1 to 0.08 BAC. For reference purposes, other countries that lowered the legal BAC in recent decades are Russia, Sweden, and Norway, which decreased it from 0.05 to 0.02 g/dL, and Poland and Japan, which did so from 0.05 to 0.03 g/dL [8,24].

There are two characteristics of the penalty structure in Chile that deserve to be noted. One is the distinction between DUI (between 0.03 and 0.079 BAC) and drunk driving (above 0.08 BAC), and the second is that the license suspension period depends on the type of injuries caused by the car crash. See Otero and Rau [13] for a complete description of the ZTL and its effects.

On 16 September 2014, Law 20,770 (EML) was enacted. This law resulted from a citizen initiative when on 21 January 2013, a nine-month-old minor, Emilia Silva, died after a driver with 0.19 g/dL of alcohol struck her parents’ car, which produced considerable media and social commotion. The EML modified the traffic law concerning the crime of DUI and driving while intoxicated. It punishes DUI and drunk drivers who cause severe injuries or death with at least one year of actual imprisonment. Additionally, it established as a crime to fleeing from the scene of an accident and refusing to submit to a blood alcohol test or breathalyzer [6].

Thus, while the ZTL reduces the legal driving BAC level and increased the license revocation period for DUI and drunk driving, EML increased the penalties, including at least one year of actual imprisonment, for DUI and drunk drivers who cause severe injuries or deaths. Since EML only changed the penalty structure, we are able to place particular focus on the deterrence effect of including prison sentences, absent the influence of other changes that could be confound with the effect of increasing penalties.

Finally, it is worth mentioning that in Chile, individuals must be 18 years or older to drive alone. There is a special permit for teens at 17 that allows them to drive when accompanied by an adult driver. Additionally, buying alcohol is legal for those 18 years of age or older.

## 3. Materials and Methods

### 3.1. Data and Descriptive Statistics

We use data from Carabineros de Chile, the national police force, as our main source of information. We have administrative records on all traffic accidents in Chile from January 2009 to March 2018. With these records, we obtain the date and place of the accident, number and severity of injuries, number of deaths if any and the accident’s cause. Of the 678,963 total traffic accidents, 48,019 were alcohol-related, including DUI and drunk driving. In this regard, it is important to clarify two points. First, even though a car accident could have more than one cause, the police officer at the scene classifies the accident according to its root or main cause. Rizzi and Fariña [25] argue that this may be done to establish the legal responsibilities of drivers involved in accidents. Second, the severity of injuries that results from a car accident is defined objectively by the legal framework. An injury is defined as severe if the injured individual cannot work for at least 30 days after the accident, as moderate if it is between 5 and 30 days, and minor for less than five days.

We collapse our data into a longitudinal dataset, by month and geographical region, to avoid an excess of zeros in injuries and deaths in many of the accidents. With a total of 15 regions and 111 months, we have a panel database of 1665 observations. To control for relevant economic activity we use gasoline sales (in thousands of m${}^{3}$ per region and month) as a proxy variable, obtained from the National Energy Commission. An increase in economic activity translates into increases in the aggregated demand for goods and services in the economy, which raises gasoline sales. An additional concern is related to changes in traffic accidents that could be explained by changes in law enforcement. For this reason, we also control for enforcement in our models using police stops per region/month, information obtained from the national police force. Moreover, to avoid simultaneity issues, we use lagged police stops, which accounts for a loss of 15 observations. We merge gasoline sales, police stops and our main outcomes of interest by region and month.

Our second source of information is Servicio Médico Legal, the Chilean legal institution of medical services. For the same period, we have access to all 358,460 BAC tests in the Metropolitan Region, including test date, BAC result, and gender of the individual being tested. We use these data at the micro level to investigate alcohol consumption as a potential channel through which both laws operate. The Metropolitan Region is one of the country’s 15 regions, and it hosts the country’s capital, Santiago. It is the most populous region, with a population of 7.9 million of the national total of 19.1 million (41%) in 2019 [26]. The Metropolitan Region is a representative sample of the country since it contains 40% of the national total of motorized vehicles and 35% of the traffic accidents, similar to its population share [27].

It is important to remark two points about the data we use. First, all our data comes from administrative records of public access. Both Carabineros de Chile and Servicio Médico Legal provided information on all traffic accidents nationwide and all BAC tests in the Metropolitan Region, respectively. In this sense, we do not use survey data nor intervene in the information gathering process. Second, there might be some degree of underreporting in traffic accidents, particularly in those considered minor ones. This fact has been well documented in the literature worldwide [28,29,30]. We believe underreporting has a small effect on our results since we focus on alcohol-related accidents, which have a higher probability of being reported, especially when they have injuries and deaths.

Table 1 presents summary statistics of all our variables. There are 407.79 traffic accidents on average per region every month, of which 28.84 (7%) are alcohol-related. These accidents result in 0.94 deaths and 28.33 injuries, of which 21.15 are minor injuries, 2.65 are moderate, and 4.53 are severe injuries. Also, the average number of monthly regional police stops and gas sales are 41.29 stops and 21.83 thousands of m${}^{3}$, respectively. Finally, from the 358,460 individuals tested, 82% of them are males, and the BAC mean level is about 0.03 g/dL.

### 3.2. Empirical Methods

We estimate negative binomial regressions for our primary outcomes: alcohol-related accidents, injuries, and deaths. Following Otero and Rau [13], our analysis of blood alcohol tests—our secondary outcome of interest—is performed by estimating generalized linear models (GLM) and censored quantile regressions.

#### 3.2.1. Negative Binomial Regressions

Our main outcomes of interest—that is, accidents, injuries, and deaths—are defined as count variables, and it is natural to assume that they follow a Poisson distribution.

We use negative binomial regressions for our count variables since, as discussed by Fridstrøm [31], these variables have variance larger than their expectation, suggesting the use of the negative binomial distribution (also known as the Poisson-gamma mixture) that allows for overdispersion. See Cameron and Trivedi [32] and Hilbe [33] for more details on count models. In this case, the conditional expectation function is given by:

(1) $$E\left[y_{i,t}|x_{i,t}\right]=\mu_{i,t}=\exp\left[\alpha +\eta t+\beta_{1}{\mathrm{ZTL}}_{t}+\beta_{2}{\mathrm{ZTL}}_{t}\times \left(t-t_{1}\right)+\delta_{1}{\mathrm{EML}}_{t}+\delta_{2}{\mathrm{EML}}_{t}\times \left(t-t_{2}\right)+z_{i,t}\gamma \right],$$

where $x_{i,t}$ is a row vector that represents all the covariates included in the model for geographical region *i* in month *t*. Covariate *t* is a monthly linear trend and ${\mathrm{ZTL}}_{t}=1_{\left[t\geq t_{1}\right]}$ and ${\mathrm{EML}}_{t}=1_{\left[t\geq t_{2}\right]}$ are indicator functions for each law, with $t_{1}$ and $t_{2}$ being the enactment months for the ZTL and EML, respectively. We also include interactions for both indicator functions with *t* to assess the dynamics of each law’s effects. For ease of analysis, trends in interactions are centered by subtracting the respective month of enactment. $z_{i,t}$ is a row vector of other region-month control variables such as police stops, gas sales, regional fixed effects and month-of-the-year fixed effects. Finally, γ is the corresponding column vector of parameters. In this model, the conditional variance is given by $V\left[y_{i,t}|x_{i,t}\right]=\mu_{i,t}\left(1+\theta \mu_{i,t}\right)$, where θ>0 is the overdispersion parameter.

Under this specification, we can estimate the following dynamic effects:

- Marginal effect of the ZTL in month *t*:
  (2) $$\Delta \%E\left[y_{i,t}|x_{i,t}\right]=\exp\left[\beta_{1}+\beta_{2}\left(t-t_{1}\right)\right]-1,$$
  that is, the effect of the ZTL, $t-t_{1}$ months after its enactment; with $t_{1}\leq t<t_{2}$.
- Marginal effect of EML in month *t*:
  (3) $$\Delta \%E\left[y_{i,t}|x_{i,t}\right]=\exp\left[\delta_{1}+\delta_{2}\left(t-t_{2}\right)\right]-1,$$
  that is, the effect of the EML, $t-t_{2}$ months after its enactment, with $t\geq t_{2}$.

See Appendix A for a detailed derivation of these effects.

#### 3.2.2. Generalized Linear Models

We use generalized linear models (GLMs) to estimate the effects of the ZTL and EML on the BAC distribution. To address the nonnegative nature of BAC, we consider the following GLM with a Gaussian family and log link function:

(4) $$E\left[y_{i,t}|x_{i,t}\right]=g^{-1}\left[\alpha +\beta_{1}{\mathrm{ZTL}}_{t}+\delta_{1}{\mathrm{EML}}_{t}+\gamma {\mathrm{male}}_{i}+\beta_{2}{\mathrm{ZTL}}_{t}\times {\mathrm{male}}_{i}+\delta_{2}{\mathrm{EML}}_{t}\times {\mathrm{male}}_{i}\right],$$

where ${\mathrm{male}}_{i}$ is a dummy variable for male and interaction terms between both laws and gender are included since it has been reported that laws that lower the legal blood alcohol content for driving have larger effects for males [34]. Other demographic variables, such as age, have also been studied in the literature [34,35]. However, in our data, gender is the only demographic variable provided by Servicio Médico Legal (our second source of information).

For the link function g(·), we use two different specifications: (i) an identity link function with Gaussian residuals, which is equivalent to a linear model, and (ii) a log link function with Gaussian residuals since BAC ≥0. For both specifications, we also include month-of-the-year fixed effects and a daily linear trend.

#### 3.2.3. Censored Quantile Regressions

Finally, to evaluate a potential heterogeneous response to both laws at different quantiles of the BAC distribution, we estimate quantile regressions with left-censoring at 0, following Koenker and Bassett [36] and Powell [37]. Our specification for quantile τ is given by:

(5) $$Q_{\tau }\left[y_{i,t}|x_{i,t}\right]=\alpha \left(\tau \right)+\beta_{1}\left(\tau \right){\mathrm{ZTL}}_{t}+\delta_{1}\left(\tau \right){\mathrm{EML}}_{t}+\gamma \left(\tau \right){\mathrm{male}}_{i}+\beta_{2}\left(\tau \right){\mathrm{ZTL}}_{t}\times {\mathrm{male}}_{i}+\delta_{2}\left(\tau \right){\mathrm{EML}}_{t}\times {\mathrm{male}}_{i},$$

where we also include a daily linear trend. We estimate the effect of both laws for the 85th, 90th, and 95th quantiles of the distribution, following the discussion in Section 3.1.

## 4. Results

All our analysis was conducted using Stata 16. For statistical significance we use 5% as the reference level.

### 4.1. Accidents, Injuries and Deaths

**Figure 1:**
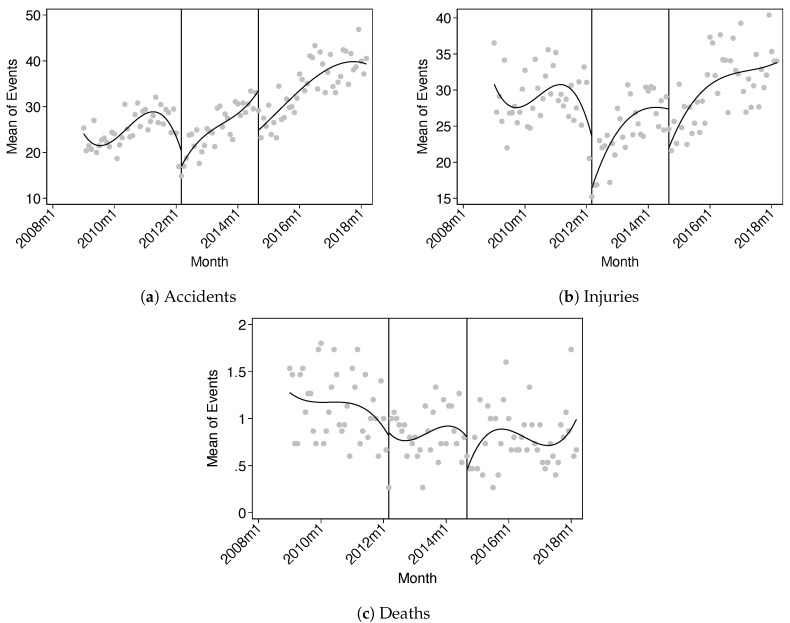
Plots for alcohol-related accidents, injuries and deaths, along with cubic fits. Each dot represents the regional average of events in a given month. The first and second vertical lines represent the enactment month of the Zero Tolerance Law (ZTL) and Emilia’s Law (EML), respectively.

Before presenting the results of our negative binomial regression models, we analyze the evolution of our main outcomes of interest. Figure 1 depicts the changes over time of alcohol-related accidents, injuries, and deaths in panels (a–c), respectively. Each dot represents the regional average of events in a given month. In addition, lines interrupted by the enactment month of each law are included to capture the overall trend. These lines are estimated by ordinary least squares (OLS) where we include as independent variables a cubic polynomial of time.

For accidents and injuries, we observe a negative jump in the series immediately after ZTL and EML enactment, suggesting a significant short-run decrease. For the case of deaths, we see an overall decreasing trend without clear jumps in either of the enactment months.

Table 2 presents the estimation results for the parameters of the negative binomial regressions in Equation (1). The ZTL-related coefficients are very close to those estimated by Otero and Rau [13]. In particular, we find negative and significant coefficients for the post-ZTL variable and positive and significant coefficients for the interaction term for accidents and injuries. This indicates that the ZTL effects vanish with time. The coefficients for the EML dummies are also negative and significant (except for severe injuries that is nonsignificant), although of smaller magnitude than the ZTL dummy coefficients. Interestingly, we find negative coefficients for the $\mathrm{EML}\times (t-t_{2})$ interactions, suggesting a reinforcement of the law’s effect over time, at least for accidents and minor injuries. For deaths, however, we find no significant results. Since deaths from alcohol-related accidents are less frequent than injuries, and to avoid an excess zeros problem, Appendix B provides the results of zero-inflated negative binomial regression for deaths, obtaining similar results.

The estimated coefficients for the control variables are statistically significant in some specifications. For example, while gas sales are positively associated with alcohol-related car accidents, all and minor injuries, they are not statistically significantly associated with moderate injuries and deaths.

Goodness of fit measures are presented for all our primary outcomes and different models. In all cases, the Wald chi-square statistic and corresponding *p*-value confirm our models’ statistical significance. The null hypothesis that all coefficients are equal to zero is rejected at 1% level. We also present pseudo-$R^{2}$, with similar results to the previous literature [13,18].

To better understand the dynamic patterns of the two policies, Table 3 shows a summary of the effects of each law two years after enactment in six-month intervals, using Equations (2) and (3). As shown, while the ZTL effects decrease in magnitude, the EML effect reinforces after two years. For example, in alcohol-related accidents, the ZTL effects range from −33.66% to −11.90%, and those of EML range from −21.88% to −30.98%.

To assess the robustness of our results, we estimate two additional models for all of our primary outcomes. First, we consider generalized Poisson regressions as an alternative specification to the negative binomial distribution assumption. Generalized Poisson models allow for under- and overdispersion in the outcome variable. Second, given that both laws were enacted around mid-month, it could be argued that our post dummy variables should be equal to one from the first month of full enforcement (i.e., April 2012 and October 2014 for the ZTL and EML, respectively). To address this concern, we estimate our models as in Equation (1) but change both dummy variables accordingly. Our main results remain similar in both cases, and the dynamics of the effects continue unchanged. The results for both models are in Appendix C. We now turn to the results of our secondary outcome of interest.

### 4.2. Blood Alcohol Content

We begin by presenting visual evidence about the potential effects of both laws on BAC tests. To do so, we estimate BAC densities for three relevant periods: (i) between January 2009 and February 2012 (i.e., no law in place), (ii) between March 2012 and August 2014 (i.e., only the ZTL in place), and (iii) between September 2014 and March 2018 (i.e., both laws in place). Density estimations use kernel functions to represent the probability density function of a random variable nonparametrically. Two essential elements in the density estimation are the kernel function and the bandwidth choice that determine the resulting density’s smoothness. We follow Silverman’s approach, which implies an Epanechnikov kernel function and a plugin estimator for the bandwidth [39]. See Fox and Long [40] for more details on density plots.

**Figure 2:**
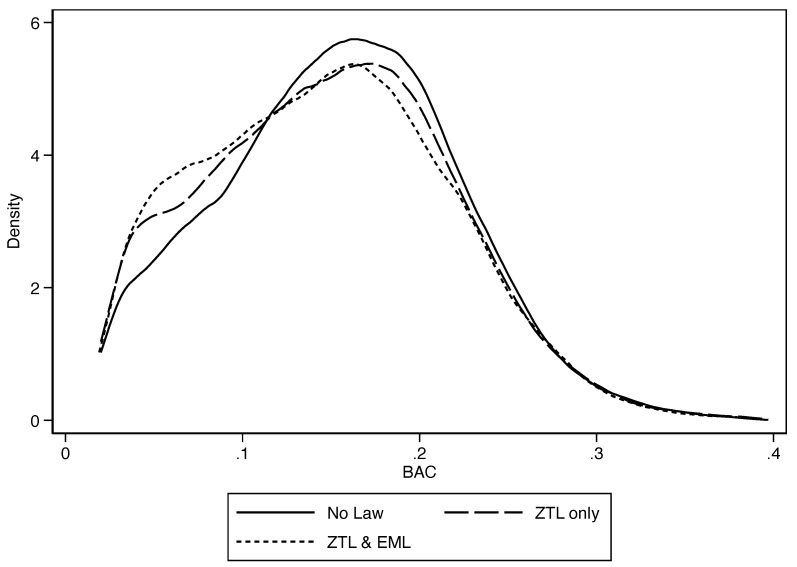
Kernel density plots for blood alcohol content (BAC) tests. For ease of exposition, only 0<BAC≤0.4 are included. The numbers of observations are 24,255, 17,431 and 22,191 for the no law, ZTL only and ZTL & EML periods, respectively. The Epanechnikov kernel is used, and bandwidth selection follows Silverman [39].

Figure 2 depicts the BAC distribution at higher quantiles only, as 82.17% of our data contain zeros. There seems to be no difference in density in the top quantiles across the three curves (from approximately the 98th quantile). However, below the 95th quantile (i.e., below 0.2 g/dL), the BAC distribution when the ZTL and both laws were in force shifts to the left compared to its distribution before their passage.

Table 4 presents the estimation results of Equation (4) for both specifications discussed in Section 3.2.2. Columns (1) and (3) omit the interaction term with gender and indicate that both laws have a negative and significant coefficient, with the ZTL coefficient more than doubling that of EML. When we add the gender interaction with both laws in columns (2) and (4), the coefficient patterns differ between the ZTL and EML. For the ZTL, we find that the dummy variables’ coefficients are much smaller than in columns (1) and (3), with the ZTL×Male interactions absorbing most of the effect. These results are different in the case of EML. In that case, the interaction terms EML×Male are nonsignificant, while the dummy coefficients remain close to those in columns (1) and (3), suggesting that EML’s effect on BAC is the same regardless of gender. All our models are statistically significant as shown by goodness of fit measures.

Finally, Table 5 presents the results from censored quantile regressions of Equation (5). In line with the results in Table 4, the male coefficients are positive and significant in all three higher quantiles evaluated. Regarding the post-ZTL and post-EML coefficients, there is no significant effect. However, when analyzing the interaction terms’ coefficients, we find a negative and significant effect of ZTL×Male in the 85th and 90th quantiles. For the EML case, only the 85th quantile interaction is significant and approximately one-sixth of its ZTL counterpart.

## 5. Discussion

Our results reveal a significant short-term reduction in alcohol-related accidents and injuries for both laws. For the ZTL, the effects are short-lived, as found previously by other authors for different BAC laws [13,41,42]. A common explanation for this lack of impact in the long run is the limited enforcement of the law after its enactment. However, for EML, the magnitude of the results does not decrease with time. Hence, in the Chilean context, the inclusion of an actual prison sentence may have a sustained effect on alcohol-related accidents and injuries.

While we find significant results for alcohol-related accidents and injuries after the enactment of both laws, we do not find a significant effect of the ZTL or EML on deaths. As noted by Otero and Rau [13], such a result may be due to a lack of statistical power given the small number of deaths. However, this lack of effect is consistent with previous findings in the international literature [43,44,45]. As discussed by Grant [44] and Otero and Rau [13], one explanation is simply that these types of laws affect drivers involved in non-fatal accidents. Given that drunk drivers cause the vast majority of deaths in alcohol-related accidents, our BAC results provide evidence along these lines.

According to the Carabineros de Chile data, between 2012 and 2017, 79% of deaths in alcohol-related accidents were due to drunk driving (i.e., 0.08 BAC and above) and the rest to DUI (0.03–0.079 BAC). Interestingly, when analyzing the effects of both laws on BAC, our results show a significant impact up to the 90th quantile. Hence, heavy drinkers who drive (above the 90th quantile of the BAC distribution) seem to not change their alcohol consumption, in contrast to drivers who drink less.

Importantly, the ZTL and EML seem to affect the blood alcohol content of drivers differently. First, the ZTL shows stronger effects than EML; the coefficients are larger in magnitude and affect higher quantiles of the BAC distribution. Second, while the ZTL affects male drivers only, EML affects both males and females. The result of BAC laws affecting only males has been previously found in Europe by Albalate [34] and in the U.S. by Eisenberg [46]. However, our result of increasing the penalties for DUI and drunk driving on BAC is new. An actual jail sentence affects male and female drivers equally.

Certainly, the deterrence effect of any law may be influenced by its enforcement and severity. The Chilean case offers an interesting opportunity to compare two policies with a similar enforcement level and different harshness in their punishments. While the ZTL considers lengthened license suspension periods, the EML prescribes actual prison sentences.

Our results are consistent with previous findings in the literature. In particular, many authors find that stiffening the penalties for drunk driving, including actual jail time, has no long-term effects on alcohol-related fatalities and other outcomes for various reasons [14,15,16]. Some authors argue that drunk driving laws should focus on increasing the probability of detection for law violators rather than increasing the severity of the penalty [15]. Other authors state that penalties, including prison sentences, are not severe enough [16]. Finally, there might be other factors affecting alcohol-related traffic fatalities, such as high taxes on alcohol, marketing restrictions, and inexpensive transportation alternatives [17].

This paper provides evidence on the effects of introducing actual imprisonment for drunk drivers responsible for severe injuries or fatal crashes. More importantly, the length of the jail sentence under EML of at least one year can be considered harsh relative to other countries’ punishments. Nevertheless, we found no effect on alcohol-related traffic fatalities. One limitation of our study is that we have a post-intervention period of EML of four years only, and the impact of this type of intervention may take longer. More research is needed to assess the effects of drunk driving laws in the long run and fully understand how they work. This might shed light on how they could deter heavy drinkers from driving.

## Figures and Tables

**Table 1:** Summary Statistics.

	Mean	SD	Min	Max
Data from Carabineros de Chile:				
All Accidents	407.79	528.67	6.00	3225.00
Alcohol Accidents	28.84	25.40	0.00	152.00
All Injuries	28.33	23.04	0.00	124.00
Minor Injuries	21.15	17.70	0.00	104.00
Moderate Injuries	2.65	2.74	0.00	17.00
Severe Injuries	4.53	4.28	0.00	27.00
Deaths	0.92	1.36	0.00	16.00
Police Stops	41.29	33.26	1.78	168.04
Data from the National Energy Commission:				
Gas Sales	21.83	32.93	1.30	174.69
Data from Servicio Médico Legal:				
BAC Test	0.03	0.06	0.00	0.64
Male Proportion	0.82	0.39	-	-

Notes: SD stands for Standard Deviation. Data from Carabineros de Chile and the National Energy Commission is monthly and regional and we have 1665 observations. Data from Servicio Médico Legal is for the Metropolitan Region only and at the individual BAC test level. In this case we have 358,460 observations.

**Table 2:** Negative Binomial Regressions.

	Accidents	Injuries				Deaths
		All	Minor	Moderate	Severe	
	**(1)**	**(2)**	**(3)**	**(4)**	**(5)**	**(6)**
Trend	0.0063 ***	0.0006	0.0006	0.0012	−0.0018	−0.0075
	(0.0014)	(0.0017)	(0.0018)	(0.0029)	(0.0023)	(0.0046)
Post ZTL	−0.4103 ***	−0.4071 ***	−0.4250 ***	−0.4295 ***	−0.2979 ***	−0.2407
	(0.0475)	(0.0612)	(0.0609)	(0.1079)	(0.0854)	(0.1601)
Post ZTL × Trend	0.0118 ***	0.0143 ***	0.0161 ***	0.0107 *	0.0117 ***	0.0118
	(0.0026)	(0.0032)	(0.0033)	(0.0056)	(0.0044)	(0.0087)
Post EML	−0.2469 ***	−0.2079 ***	−0.2249 ***	−0.2295 **	−0.0788	−0.2400
	(0.0456)	(0.0554)	(0.0591)	(0.0961)	(0.0750)	(0.1621)
Post EML × Trend	−0.0052 **	−0.0064 **	−0.0075 **	−0.0077	−0.0060	0.0007
	(0.0025)	(0.0031)	(0.0031)	(0.0054)	(0.0042)	(0.0087)
Police Stops	0.0011 **	−0.0004	−0.0001	−0.0017	−0.0003	0.0030 *
	(0.0005)	(0.0006)	(0.0006)	(0.0011)	(0.0009)	(0.0016)
Gas Sales	0.0086 ***	0.0074 ***	0.0083 ***	0.0030	0.0061 *	0.0073
	(0.0019)	(0.0022)	(0.0023)	(0.0041)	(0.0032)	(0.0046)
Constant	1.8500 ***	2.1930 ***	1.8680 ***	−0.0034	0.4859 ***	−1.1388 ***
	(0.0953)	(0.1126)	(0.1200)	(0.1862)	(0.1412)	(0.2881)
ln(θ)	−2.6346 ***	−1.9314 ***	−1.9304 ***	−1.8503 ***	−2.3507 ***	−1.5758 ***
	(0.0977)	(0.0735)	(0.0763)	(0.1405)	(0.1373)	(0.2368)
Observations	1650	1650	1650	1650	1650	1650
Pseudo-$R^{2}$	0.1989	0.1505	0.1550	0.1429	0.1779	0.1446
Wald test	10,169.41	5350.44	4869.16	1190.76	2066.18	590.25
*p*-value	0.0000	0.0000	0.0000	0.0000	0.0000	0.0000

Notes: Robust standard errors in parentheses. All models include regional fixed effects and month-of-the-year fixed effects. θ represents the overdispersion parameter. Pseudo-$R^{2}$ as in McFadden [38]. *** *p* < 0.01, ** *p* < 0.05, * *p* < 0.1.

**Table 3:** Percentage Change in Accidents and Injuries over Time.

Months after Enactment	0	6	12	18	24
Panel A: Marginal effect of ZTL					
Accidents	−33.66	−28.78	−23.55	−17.93	−11.90
Injuries	−33.45	−27.50	−21.02	−13.97	−6.28
Panel B: Marginal effect of EML					
Accidents	−21.88	−24.26	−26.57	−28.81	−30.98
Injuries	−18.77	−21.82	−24.75	−27.57	−30.29

Note: Calculations follow Equations (2) and (3).

**Table 4:** Generalized Linear Models: Marginal Effects.

	OLS		GLM	
	(1)	(2)	(3)	(4)
Post ZTL	−0.0067 ***	−0.0013 **	−0.0077 ***	0.0002
	(0.0004)	(0.0005)	(0.0005)	(0.0011)
Post EML	−0.0023 ***	−0.0023 ***	−0.0028 ***	−0.0032 ***
	(0.0004)	(0.0005)	(0.0004)	(0.0009)
Male	0.0184 ***	0.0228 ***	0.0187 ***	0.0213 ***
	(0.0002)	(0.0004)	(0.0002)	(0.0004)
Post ZTL × Male		−0.0066 ***		−0.0080 ***
		(0.0005)		(0.0011)
Post EML × Male		−0.0000		0.0005
		(0.0005)		(0.0010)
Observations	358,460	358,460	358,460	358,460
Pseudo-$R^{2}$	0.0148	0.0151	0.0152	0.0153
Wald test	8391.13	8536.22	4760.68	4935.85
*p*-value	0.0000	0.0000	0.0000	0.0000

Notes: Robust standard errors in parentheses. All models include month-of-the-year fixed effects and a daily linear trend. Pseudo-$R^{2}$ as in McFadden [38]. *** *p* < 0.01, ** *p* < 0.05, * *p* < 0.1.

**Table 5:** Censored Quantile Regressions.

	Quantile		
	85th	90th	95th
Post ZTL	−0.0000	−0.0000	0.0034
	(0.0000)	(0.0039)	(0.0407)
Post EML	0.0000	−0.0000	−0.0162
	(0.0000)	(0.0039)	(0.0229)
Male	0.1210 ***	0.1640 ***	0.0923 ***
	(0.0011)	(0.0008)	(0.0048)
Post ZTL × Male	−0.0300 ***	−0.0170 ***	−0.0176
	(0.0019)	(0.0041)	(0.0198)
Post EML × Male	−0.0050 **	−0.0010	0.0080
	(0.0025)	(0.0043)	(0.0239)
Constant	0.0000	0.0000	0.1108 ***
	(0.0000)	(0.0000)	(0.0053)
Observations	358,460	358,460	358,460

Notes: Robust standard errors in parentheses. The model also includes a daily linear trend. *** *p* < 0.01, ** *p* < 0.05, * *p* < 0.1. Goodness of fit not available for censored quantile regressions.

## Abbreviations

The following abbreviations are used in this manuscript:

ZTL	Zero Tolerance Law
EML	Emilia’s Law
BAC	Blood Alcohol Content
DUI	Driving Under the Influence
GLM	Generalized Linear Model
OLS	Ordinary Least Squares

## Appendix A. Derivation of Dynamic Effects

Rewrite $E\left[y_{i,t}|x_{i,t}\right]$ as $E\left[y_{i,t}|{\mathrm{ZTL}}_{t}=k,{\mathrm{EML}}_{t}=k,x_{-d,i,t}\right]$ with k∈{0,1} and where $x_{-d,i,t}$ is the row vector that represents all regressors except the dummy variables ${\mathrm{ZTL}}_{t}$ and ${\mathrm{EML}}_{t}$. Following Equation (1), consider the following two dynamic effects:

1. Marginal effect of ZTL in month *t*.
  The change in $E\left[y_{i,t}|x_{i,t}\right]$ between no law and ZTL can be written as:
  (A1) $$\begin{array}{cc} \; & E\left[y_{i,t}|{\mathrm{ZTL}}_{t}=1,{\mathrm{EML}}_{t}=0,x_{-d,i,t}\right]-E\left[y_{i,t}|{\mathrm{ZTL}}_{t}=0,{\mathrm{EML}}_{t}=0,x_{-d,i,t}\right]\\ \; & =\exp\left[\alpha +\eta t+\beta_{1}+\beta_{2}\left(t-t_{1}\right)+z_{i,t}\gamma \right]-\exp\left[\alpha +\eta t+z_{i,t}\gamma \right]\\ \; & =\exp\left[\alpha +\eta t+z_{i,t}\gamma \right]\cdot \left\{\exp\left[\beta_{1}+\beta_{2}\left(t-t_{1}\right)\right]-1\right\} \end{array}$$
  Then, the corresponding percentage change is given by:
  (A2) $$\begin{array}{cc} \Delta \%E\left[y_{i,t}|x_{i,t}\right]\; & =\frac{E\left[y_{i,t}|{\mathrm{ZTL}}_{t}=1,{\mathrm{EML}}_{t}=0,x_{-d,i,t}\right]-E\left[y_{i,t}|{\mathrm{ZTL}}_{t}=0,{\mathrm{EML}}_{t}=0,x_{-d,i,t}\right]}{E\left[y_{i,t}|{\mathrm{ZTL}}_{t}=0,{\mathrm{EML}}_{t}=0,x_{-d,i,t}\right]}\\ \; & =\exp\left[\beta_{1}+\beta_{2}\left(t-t_{1}\right)\right]-1 \end{array}$$
  as in Equation (2).
2. Marginal effect of EML in month *t*.
  The change in $E\left[y_{i,t}|x_{i,t}\right]$ between the ZTL and EML can be written as:
  (A3) $$\begin{array}{cc} \; & E\left[y_{i,t}|{\mathrm{ZTL}}_{t}=1,{\mathrm{EML}}_{t}=1,x_{-d,i,t}\right]-E\left[y_{i,t}|{\mathrm{ZTL}}_{t}=1,{\mathrm{EML}}_{t}=0,x_{-d,i,t}\right]\\ \; & =\exp\left[\alpha +\eta t+\beta_{1}+\beta_{2}\left(t-t_{1}\right)+\delta_{1}+\delta_{2}\left(t-t_{2}\right)+z_{i,t}\gamma \right]\\ \; & \;-\exp\left[\alpha +\eta t+\beta_{1}+\beta_{2}\left(t-t_{1}\right)+z_{i,t}\gamma \right]\\ \; & =\exp\left[\alpha +\eta t+\beta_{1}+\beta_{2}\left(t-t_{1}\right)+z_{i,t}\gamma \right]\cdot \left\{\exp\left[\delta_{1}+\delta_{2}\left(t-t_{2}\right)\right]-1\right\} \end{array}$$
  Then, the corresponding percentage change is given by:
  (A4) $$\begin{array}{cc} \Delta \%E\left[y_{i,t}|x_{i,t}\right]\; & =\frac{E\left[y_{i,t}|{\mathrm{ZTL}}_{t}=1,{\mathrm{EML}}_{t}=1,x_{-d,i,t}\right]-E\left[y_{i,t}|{\mathrm{ZTL}}_{t}=1,{\mathrm{EML}}_{t}=0,x_{-d,i,t}\right]}{E\left[y_{i,t}|{\mathrm{ZTL}}_{t}=1,{\mathrm{EML}}_{t}=0,x_{-d,i,t}\right]}\\ \; & =\exp\left[\delta_{1}+\delta_{2}\left(t-t_{2}\right)\right]-1 \end{array}$$
  as in Equation (3).

## Appendix B. Zero-Inflated Negative Binomial Regression

Deaths from alcohol-related accidents were null in 896 out of our 1665 (53.81%) observations. This feature of the data might result in our models predicting fewer zeros than observed, an issue known as *excess zeros* as discussed by Cameron and Trivedi [32]. Zero-inflated models add an additional component to the base regression to inflate the probability of zero. For the particular case of the zero-inflated negative binomial regression, the conditional expectation function is given by:

(A5) $$E\left[y_{i,t}|x_{i,t}\right]=\left(1-\pi_{i,t}\right)\mu_{i,t}$$

where $\mu_{i,t}$ is the negative binomial conditional expectation function as in Equation (1) and $\pi_{i,t}$ is the inflation component that can be represented by a binary outcome model. In particular, we use a logit link function with the monthly linear trend, both dummies, both interactions, police stops, gas sales and alcohol-related accidents as independent variables.

Table A1 presents the estimation results, with coefficients in column (1) similar to those from our negative binomial model presented in column (6) of Table 2.

**Table A1:** Zero-inflated Negative Binomial Regression.

	Negative Binomial	Logit (Inflate)
	**(1)**	**(2)**
Trend	−0.0076	0.0305
	(0.0047)	(0.0522)
Post ZTL	−0.2364	−2.6494 *
	(0.1609)	(1.4847)
Post ZTL × Trend	0.0119	0.1606
	(0.0087)	(0.1060)
Post EML	−0.2410	−0.9074
	(0.1648)	(2.0402)
Post EML × Trend	−0.0011	−0.2305 **
	(0.0087)	(0.1005)
Police Stops	0.0028 *	0.1731 *
	(0.0016)	(0.1006)
Gas Sales	0.0065	−0.4890 ***
	(0.0046)	(0.1582)
Alcohol Accidents		−0.9134 **
		(0.4255)
Constant	−0.8052 ***	3.3788 **
	(0.3062)	(1.3558)
ln(θ)	−1.7136 ***	
	(0.2508)	
Observations	1650	
Pseudo-$R^{2}$	0.1666	
Wald test	407.47	
*p*-value	0.0000	

Notes: Robust standard errors in parentheses. Negative binomial equation includes regional fixed effects and month-of-the-year fixed effects. θ represents the overdispersion parameter. Pseudo-$R^{2}$ as in McFadden [38]. *** *p* < 0.01, ** *p* < 0.05, * *p* < 0.1.

## Appendix C. Robustness Checks

### Appendix C.1. Generalized Poisson Regressions

Table A2 presents the results of the generalized Poisson regressions as an alternate specification. The results are similar to those presented in Table 2 for negative binomial regressions.

**Table A2:** Generalized Poisson Regression.

	Accidents	Injuries				Deaths
		All	Minor	Moderate	Severe	
	**(1)**	**(2)**	**(3)**	**(4)**	**(5)**	**(6)**
Trend	0.0062 ***	0.0007	0.0005	0.0011	−0.0018	−0.0074
	(0.0015)	(0.0019)	(0.0020)	(0.0029)	(0.0024)	(0.0046)
Post ZTL	−0.4112 ***	−0.4115 ***	−0.4290 ***	−0.4306 ***	−0.2985 ***	−0.2405
	(0.0487)	(0.0658)	(0.0637)	(0.1081)	(0.0854)	(0.1602)
Post ZTL × Trend	0.0119 ***	0.0141 ***	0.0162 ***	0.0107 *	0.0117 ***	0.0117
	(0.0026)	(0.0034)	(0.0034)	(0.0056)	(0.0044)	(0.0087)
Post EML	−0.2547 ***	−0.2180 ***	−0.2337 ***	−0.2314 **	−0.0784	−0.2389
	(0.0473)	(0.0598)	(0.0621)	(0.0963)	(0.0750)	(0.1622)
Post EML × Trend	−0.0047 *	−0.0055 *	−0.0069 **	−0.0076	−0.0060	0.0007
	(0.0025)	(0.0033)	(0.0032)	(0.0055)	(0.0042)	(0.0087)
Police Stops	0.0009 *	−0.0005	-0.0001	−0.0017	−0.0003	0.0030 *
	(0.0005)	(0.0007)	(0.0007)	(0.0011)	(0.0009)	(0.0016)
Gas Sales	0.0097 ***	0.0086 ***	0.0095 ***	0.0032	0.0061 *	0.0072
	(0.0021)	(0.0025)	(0.0026)	(0.0042)	(0.0032)	(0.0046)
Constant	1.8471 ***	2.1784 ***	1.8581 ***	−0.0043	0.4849 ***	−1.1389 ***
	(0.0954)	(0.1149)	(0.1215)	(0.1862)	(0.1413)	(0.2881)
ln(θ)	−3.8199 ***	−3.2158 ***	−3.1509 ***	−2.7129 ***	−3.2387 ***	−2.3911 ***
	(0.0838)	(0.0636)	(0.0648)	(0.1274)	(0.1229)	(0.2230)
Observations	1650	1650	1650	1650	1650	1650
Pseudo-$R^{2}$	0.2026	0.1532	0.1603	0.1530	0.1898	0.1557
Wald test	9432.48	5029.93	4598.63	1189.11	2059.08	589.39
*p*-value	0.0000	0.0000	0.0000	0.0000	0.0000	0.0000

Notes: Robust standard errors in parentheses. All models include regional fixed effects and month-of-the-year fixed effects. θ represents the dispersion parameter. Pseudo-$R^{2}$ as in McFadden [38]. *** *p* < 0.01, ** *p* < 0.05, * *p* < 0.1.

### Appendix C.2. First Full Month of Enforcement

Table A3 presents the results of the negative binomial regressions with the dummies ${\mathrm{ZTL}}_{t}$ and ${\mathrm{EML}}_{t}$ taking the value of 1 from April 2012 and October 2014, respectively. The results are similar to those presented in Table 2.

**Table A3:** Negative Binomial Regression.

	Accidents	Injuries				Deaths
		All	Minor	Moderate	Severe	
	**(1)**	**(2)**	**(3)**	**(4)**	**(5)**	**(6)**
Trend	0.0043 ***	−0.0017	−0.0016	−0.0009	−0.0035	−0.0099 **
	(0.0015)	(0.0017)	(0.0018)	(0.0029)	(0.0022)	(0.0044)
Post ZTL	−0.3117 ***	−0.2807 ***	−0.3036 ***	−0.3156 ***	−0.2060 **	−0.0689
	(0.0484)	(0.0624)	(0.0620)	(0.1070)	(0.0853)	(0.1548)
Post ZTL × Trend	0.0112 ***	0.0129 ***	0.0148 ***	0.0090 *	0.0113 ***	0.0078
	(0.0026)	(0.0032)	(0.0033)	(0.0055)	(0.0044)	(0.0083)
Post EML	−0.2106 ***	−0.1489 ***	−0.1669 ***	−0.1654 *	−0.0526	−0.1172
	(0.0464)	(0.0561)	(0.0599)	(0.0964)	(0.0756)	(0.1606)
Post EML × Trend	−0.0025	−0.0031	−0.0043	−0.0044	−0.0040	0.0062
	(0.0025)	(0.0031)	(0.0032)	(0.0054)	(0.0043)	(0.0086)
Police Stops	0.0010 **	−0.0004	−0.0002	−0.0018	−0.0003	0.0029 *
	(0.0005)	(0.0006)	(0.0006)	(0.0011)	(0.0009)	(0.0016)
Gas Sales	0.0085 ***	0.0073 ***	0.0082 ***	0.0030	0.0059 *	0.0070
	(0.0019)	(0.0022)	(0.0023)	(0.0041)	(0.0031)	(0.0046)
Constant	1.8840 ***	2.2332 ***	1.9064 ***	0.0320	0.5167 ***	−1.0962 ***
	(0.0956)	(0.1126)	(0.1199)	(0.1864)	(0.1408)	(0.2875)
ln(θ)	−2.5939 ***	−1.9083 ***	−1.9078 ***	−1.8280 ***	−2.3415 ***	−1.5643 ***
	(0.0949)	(0.0727)	(0.0754)	(0.1381)	(0.1365)	(0.2340)
Observations	1650	1650	1650	1650	1650	1650
Pseudo-$R^{2}$	0.1968	0.1487	0.1533	0.1417	0.1771	0.1439
Wald test	9952.24	5282.90	4775.47	1174.39	2044.36	579.03
*p*-value	0.0000	0.0000	0.0000	0.0000	0.0000	0.0000

Notes: Robust standard errors in parentheses. All models include regional fixed effects and month-of-the-year fixed effects. Pseudo-$R^{2}$ as in McFadden [38]. *** *p* < 0.01, ** *p* < 0.05, * *p* < 0.1.

## References

[1] WHO (2018). Global Health Estimates 2016: Deaths by Cause, Age, Sex, by Country and by Region, 2000–2016.

[2] Moskowitz H., Fiorentino D. (2000). A Review of the Scientific Literature Regarding the Effects of Alcohol on Driving-Related Behavior at Blood Alcohol Concentrations of 0.08 Grams per Deciliter and Lower.

[3] Levitt S.D., Porter J. (2001). How Dangerous Are Drinking Drivers. J. Polit. Econ..

[4] Killoran A., Canning U., Doyle N., Sheppard L. (2010). Review of Effectiveness of Laws Limiting Blood Alcohol Concentration Levels to Reduce Alcohol-Related Road Injuries and Deaths.

[5] IRTAD (2017). Alcohol-Related Road Casualties in Official Crash Statistics.

[6] BCN (2014). Historia de la Ley 20.770.

[7] Mann R.E., Macdonald S., Stoduto G., Bondy S., Jonah B., Shaikh A. (2001). The effects of introducing or lowering legal per se blood alcohol limits for driving: An international review. Accid. Anal. Prev..

[8] Beirness D.J., Simpson H.M. (2002). The Safety Impact of Lowering the BAC Limit for Drivers in Canada.

[9] Bernat D.H., Dunsmuir W.T., Wagenaar A.C. (2004). Effects of lowering the legal BAC to 0.08 on single-vehicle- nighttime fatal traffic crashes in 19 jurisdictions. Accid. Anal. Prev..

[10] Fell J.C., Voas R.B. (2006). The effectiveness of reducing illegal blood alcohol concentration (BAC) limits for driving: Evidence for lowering the limit to .05 BAC. J. Saf. Res..

[11] Elvik R., Høye A., Vaa T., Sørensen M. (2009). The Handbook of Road Safety Measures.

[12] Assum T. (2010). Reduction of the blood alcohol concentration limit in Norway–Effects on knowledge, behavior and accidents. Accid. Anal. Prev..

[13] Otero S., Rau T. (2017). The effects of drinking and driving laws on car crashes, injuries, and deaths: Evidence from Chile. Accid. Anal. Prev..

[14] Ross H.L., McCleary R., LaFree G. (1990). Can Mandatory Jail Laws Deter Drunk Driving–The Arizona Case, 81. J. Crim. L Criminol..

[15] Nichols J., Ross H. (1991). The effectiveness of legal sanctions in dealing with drinking drivers. J. Saf. Res..

[16] Kenkel D.S. (1993). Do drunk drivers pay their way? a note on optimal penalties for drunk driving. J. Health Econ..

[17] Ross H., Klette H. (1995). Abandonment of mandatory jail for impaired drivers in Norway and Sweden. Accid. Anal. Prev..

[18] Weinrath M., Gartrell J. (2001). Specific Deterrence and Sentence Length: The Case of Drunk Drivers. J. Contemp. Crim. Justice.

[19] Hansen B. (2015). Punishment and Deterrence: Evidence from Drunk Driving. Am. Econ. Rev..

[20] Bouffard J.A., Niebuhr N., Exum M.L. (2017). Examining Specific Deterrence Effects on DWI Among Serious Offenders. Crime Delinq..

[21] INE (2010). Estadísticas Vitales.

[22] ITF (2019). Road Safety Annual Report 2019: Chile.

[23] CDCH (2019). Anuario Estadístico de Accidentes en el Tránsito y Ferroviarios Ocurridos en Chile Durante el Año 2019.

[24] Desapriya E., Pike I., Subzwari S., Scime G., Shimizu S. (2007). Impact of lowering the legal blood alcohol concentration limit to 0.03 on male, female and teenage drivers involved alcohol-related crashes in Japan. Int. J. Inj. Control Saf. Promot..

[25] Rizzi L., Fariña P. (2013). Alcohol en Conducción y su Incidencia en la Ocurrencia de Accidentes de Tránsito con Víctimas Fatales en Chile: Falencias en las Estadísticas Nacionales. Rev. Ing. Transp..

[26] INE (2019). Estimaciones y Proyecciones de la Población de Chile 2002-2035 Totales, Regionales, Población Urbana y Rural.

[27] INE (2019). Número de Vehículos en Circulación Motorizados y No Motorizados, Según Región.

[28] Singh P., Lakshmi P.V.M., Prinja S., Khanduja P. (2018). Under-reporting of road traffic accidents in traffic police records- a cross sectional study from North India. Int. J. Community Med. Public Health.

[29] Janstrup K.H., Kaplan S., Hels T., Lauritsen J., Prato C.G. (2016). Understanding traffic crash under-reporting: Linking police and medical records to individual and crash characteristics. Traffic Inj. Prev..

[30] Dandona R., Kumar G.A., Ameer M.A., Reddy G.B., Dandona L. (2008). Under-reporting of road traffic injuries to the police: Results from two data sources in urban India. Inj. Prev..

[31] Fridstrøm L. (2015). Disaggregate Accident Frequency and Risk Modeling. A Rough Guide.

[32] Cameron A.C., Trivedi P. (2013). Regression Analysis of Count Data.

[33] Hilbe J.M. (2014). Modeling Count Data.

[34] Albalate D. (2008). Lowering Blood Alcohol Content Levels to Save Lives: The European experience. J. Policy Anal. Manag..

[35] Kaplan S., Prato C. (2007). Impact of BAC limit reduction on different population segments: A Poisson fixed effect analysis. Accid. Anal. Prev..

[36] Koenker R., Bassett G. (1978). Regression Quantiles. Econometrica.

[37] Powell J.L. (1986). Censored regression quantiles. J. Econom..

[38] McFadden D. (1977). Quantitative Methods for Analyzing Travel Behaviour of Individuals: Some Recent Developments.

[39] Silverman B.W. (1986). Density Estimation for Statistics and Data Analysis.

[40] Fox J., Long J.S. (1990). Modern Methods of Data Analysis.

[41] Ross H.L. (1973). Law, Science, and Accidents: The British Road Safety Act of 1967. J. Leg. Stud..

[42] Vingilis E., Blefgen H., Lei H., Sykora K., Mann R. (1988). An evaluation of the deterrent impact of Ontario’s 12-hour licence suspension law. Accid. Anal. Prev..

[43] Freeman D. (2007). Drunk Driving Legislation and Traffic Fatalities: New Evidence on BAC 08 Laws. Contemp. Econ. Policy.

[44] Grant D. (2010). Dead on arrival: Zero tolerance laws don’t work. Econ. Inq..

[45] Anderson D.M., Hansen B., Rees D.I. (2013). Medical Marijuana Laws, Traffic Fatalities, and Alcohol Consumption. J. Law Econ..

[46] Eisenberg D. (2003). Evaluating the effectiveness of policies related to drunk driving. J. Policy Anal. Manag..

